# Molecular identification of non-tuberculous mycobacterial species isolated from extrapulmonary samples using real-time PCR and *rpoB* sequence analysis

**DOI:** 10.1186/s13568-023-01553-8

**Published:** 2023-05-05

**Authors:** Mohammad Hashemzadeh, Aram Asarehzadegan Dezfuli, Azar Dokht Khosravi, Maryam Moradi Bandbal, Atousa Ghorbani, Mahtab Hamed, Soolmaz Khandan Dezfuli

**Affiliations:** 1 Infectious and Tropical Diseases Research Center, Health Research Institute, Ahvaz Jundishapur University of Medical Sciences, Ahvaz, Iran; 2 Department of Microbiology, Faculty of Medicine, Ahvaz Jundishapur University of Medical Sciences, Ahvaz, Iran; 3 Immunobiology Center of Pasteur Medical Laboratory, Ahvaz, Iran

**Keywords:** Nontuberculous mycobacteria, Tuberculosis, Extrapulmonary, Pulmonary, Molecular methods

## Abstract

Tuberculosis (TB) is one of the leading causes of mortality among infectious diseases and accounts for a serious health hazard wordwide. Apart from TB, the members of non-tuberculous mycobacteria (NTM), which includes around 170 species, may also cause different diseases in humans. Therefore this study aimed to investigate the distribution of NTM strains isolated from extrapulmonary (EP) samples by Real-Time PCR and PCR-sequencing methods in Southwest Iran. Three hundred and twenty-five suspected EP samples were collected from patients referred to the referral hospitals in Ahvaz, Iran. The isolates were initially screened by acid fast staining and identified by phenotypic culture and biochemical tests. The Real-Time PCR and *rpoB*- based PCR methods were performed followed by sequence analysis of *rpoB* gene. From 124 samples, 77 (62%) were positive for NTM by culture and *rpoB* sequence analysis. *M. fortuitum* was the most commonly isolated NTM in present study. In Real-Time PCR, only 69 (55.64%) isolates showed more homology with standard NTM isolates. In general, the growing trend of EPNTM infections in Iran needs specific programs and resources to get a better diagnosis. PCR sequencing is a reliable method, it can be used for definitive identification of positive cultures for identification of NTM species.

## Introduction

Tuberculosis (TB) is one of the leading causes of mortality among infectious diseases and accounts for a serious health hazard wordwide (WHO, 2022). *Mycobacterium tuberculosis* (MTB) the causative agent of TB, is mainly infecting the lungs which leads to pulmonary TB (PTB), however, in certain circumstances the bacterium may infect other organs and cause extrapulmonary TB (EPTB) with less frequency in comparison to PTB (Le**e** et al. 2015). EPTB affects organs like the genital tract, lymph nodes, skin, and joints (Fang et al. 2022). Apart from MTB, the members of non-tuberculous mycobacteria (NTM), which includes around 170 species of mycobacteria, may also cause different diseases in humans. Certain NTM species including *M. avium* complex (MAC), *M. kansasii*, and *M. abscessus* are among the most common opportunistic pathogens with the capacity to cause serious infections especially in immunocompromised hosts (Gopalaswamy et al. 2020). A major challenge in laboratories for mycobacterial clinical samples, is the differential diagnosis of TB and NTM infections, which is misleading in many cases, as both MTB and NTM species demonstrate positive results using conventional smear acid-fast staining. Thus, there is an underestimated incidence of NTM in many TB-endemic countries like Iran (Schlossberg et al. 2020). Extrapulmonary non-tuberculous mycobacterial diseases (EPNTM), constitutes about 20% of all cases of TB in Iran, as the NTM species are most commonly related to lung disease (Nasiri et al. 2018a, b). Therefore, it is important to have an early differential diagnosis between TB and NTM lung infections, since despite the identical clinical presentations, there are differences in terms of epidemiology, treatment, and prognosis (Feng et al. 2020). Meanwhile, the capability of NTM to cause infections is extensively discussed in other studies and there is a growing trend of attention to it, as these are important causes of pulmonary and extrapulmonary disease in immunosuppressed hosts such as individuals with HIV infection and renal transplant patients (Henkle et al. 2015; Lapinel et al. 2019; Song et al. 2018).

Generally, molecular methods are specific and fast methods and have a higher rate of reliable detection of MTB from EPNTM and PTB samples compared to time-consuming phenotypic methods (Nurwidya et al. 2018). These methods are also shown promising results in the detection of MTB from clinical samples with negative culture results (Razavi et al. 2018). In addition, there is a growing volume of isolation of different NTM species by the application of molecular methods in medical laboratories (Adékambi et al. 2003). Following the molecular techniques, gene sequencing such as *rpoB* and 16 S rRNA have drawn a great deal of attention for mycobacterial identification (Ghielmetti et al. 2020). Given the reliable discriminatory power, the tool is widely used by many epidemiological studies to detect the species responsible for human diseases and outbreaks as well as for taxonomic re-classification. Along with a fast and reliable differential diagnosis between MTB and NTM, the molecular techniques provide rapid accurate diagnosis, which can help with the early and appropriate therapeutic management of the patients (Adékambi et al. 2003). Hence, the aim of this study was to investigate the distribution of NTM species isolated from EP samples by Real-Time PCR and PCR-sequencing methods in Southwest Iran.

## Materials and methods

### Sampling

In total 198 EP samples suspected to NTM infection including lymph node biopsy, urine, skin legion aspiration, pleural fluid, and bone biopsy, were collected from patients admitted to referral hospitals in Ahvaz city, Southwest Iran, from the beginning to the end of year 2022. The initial proposal of the work was approved in the University high research and ethics combined committee and necessary permission for sample collection was granted.

### Phenotypic identification

All samples were subjected to phenotypic identification. For all samples smear was prepared and Ziehl–Neelsen staining (ZNS) was performed for the presence of acid fast bacilli (AFB). For cultivation, the decontamination of all samples was done as described by Kent (1985). In brief, this was done by using 4% N-Acetyl-L cysteine-sodium hydroxide with subsequent centrifugation at 3000 g for 15 min and re-suspensin of decontaminated sample in phosphate buffer. About half a milliliters aliquot of decontaminated samples was inoculated onto Lowenstein Jenson (LJ) media (Biomerieux, F-69,280 Marcy l’Etoile, France), incubated at 37 °C for 8 weeks, and examined weekly for growth. Mycobacterial isolates were identified by conventional phenotypic and biochemical tests including colony morphology, growth at 25, 37, and 42 °C, pigment production, semi-quantitative catalase test, Tween 80 hydrolysis, arylsulfatase test, heat-stable catalase (pH 7, 68 °C), urease, and nitrate reduction test (Kent 1985). Out of the total 198 suspected samples, 74 (37.3%) were identified as *Mycobacterium tuberculosis* and excluded from the study. The rest 124 (62.6%) suspected samples to NTM infection, were included in the study for definitive identification.

### DNA extraction

The mycobacterial isolates grown on LJ medium were used for extraction of genomic DNA using DNA extraction QIAamp Mini Kit (Qiagen, Hilden, Germany), according to the manufacturer’s instructions. The obtained DNA was diluted 10 fold using distilled water and the concentration was determined using a Nanodrop instrument (Thermo Fisher Scientific, Waltham, MA, USA), which was then used as a template DNA for molecular assays.

### Identification of NTM species by PCR sequencing

For NTM molecular identification, a 750-bp fragment of the *rpoB* gene was amplified using MycoF and MycoR primers (Adékambi et al. 2003). The PCR mixture was prepared in a final volume of 25 µl comprising 10X PCR buffer, 1.5 mmol of MgCl_2_, 0.2 mmol of each dNTP, 1 U/µl of *Taq* DNA Polymerase, 1 µmol of each primer, 5 µl genomic DNA (50 ng), and 18 µl sterile deionized water. The PCR products were visualized by electrophoresis on 1% agarose and the results were recorded using a gel documentation system (Protein Simple, San Jose, California, USA), after staining with DNA safe stain (Yektatajhiz, Iran). A 100 bp molecular marker was used to determine the size of produced fragments.

### Nucleotide sequencing

The amplified PCR products for each isolate were purified with the Gene JETTM Gel Extraction Kit (Fermentas, Lithuania), according to manufacturer’s guidelines. An ABI PRISM 7500 Sequence Detection System (Applied Biosystems, Foster City, CA, United States) was used to determine the sequences of the products. The sequences of the *rpoB* gene for each isolate were examined using BLAST separately, and multiple sequence alignment (MSA) was carried out on sequences and the available pertinent sequences of NTM recovered from the GenBank database, using the MEGA7 program (Saitou and Nei 1987).

### The real-time PCR method for the detection of NTM

All samples were investigated by Seegene NTM PCR test (Seegene, Soul, South Korea). Overall, 5 µl of an aliquot of the supernatant was mixed with 15 µl of master mix, which contains 10 µl×2 Anyplex PCR master mix, 2 µl 10×MTB/NTM oligonucleotide mix, and 3 µl 8- methoxypsoralen. CFX96 Touch RT-PCR Detection System (Bio-Rad Laboratories Inc., USA) was used for amplification and identification of NTM, which detects the 16 S rRNA gene. For quality control, the kit contains in-house extraction controls, positive and negative amplification, and internal control in the master mix, and we processed them in each run. The interpretation of data was done automatically by CFX Maestro software that showed the results to threshold and cutoff values. Positive and negative controls were used for quality control in each run.

### Nucleotide sequence accession numbers

The sequences for each detected NTM isolate were aligned separately and compared with all existing relevant sequences of mycobacteria recovered from GenBank database, and the sequences were deposited in GenBank under accession numbers OQ466451-OQ466527.

### Statistical analysis

Statistical Package for Social Sciences (SPSS), version 22 (IBM Inc., Armonk, New York, USA) was used to analyze the data. Data were presented as mean ± SD, frequencies, and percentages. LJ culture was used as a gold standard test, whereas ZNS and NTM RT-PCR as screening test. Specificity, sensitivity, positive predictive, and negative predictive values were measured as recommended by Standards for Reporting of Diagnostic Accuracy Studies.

## Results

Our study included 124 samples suspected of EPNTM infections. The results of phenotypic tests (ZNS, LJ culture, and biochemical tests) and molecular methods (Real-time PCR and *rpoB-*based PCR sequencing) are shown in Table 1. From 124 samples, 77 (62%) were positive for NTM by culture and biochemical tests, and *rpoB* gene sequence analysis. Among suspected samples (124), 29 (23.38%) were positive by ZNS, while culture and biochemical tests showed 47 (37.90%) samples were positive. RT-PCR revealed that 69 (55.64%) EP samples were positive for NTM species. The 77 positive samples belonged to 31 female (48.05%) and male (59.74%) patients with mean ± SD age of 47.35 ± 14.58 years. The distribution of different samples in relation to patients’ gender is presented in Table 2. The positive samples were recovered from the following samples: lymph node (n = 39, 50.64%), pleural fluid (n = 23, 29.87%), skin lesion aspiration (n = 10, 12.98%), urine (n = 3, 3.89%), and bone biopsy (n = 2, 2.59%) [Table 2]. The analyzed EP positive samples were collected from different hospitals in Ahvaz city and the majority of isolates were originated from Razi (n = 43/77, 55.84%) and Golestan (n = 34 /77, 44.15%) teaching hospitals. Based on patients’ files, the most frequent past medical history was human immunodeficiency virus (HIV) infection (n = 51/77, 66.23%), and TB (n = 21/77, 27.27%). Other medical histories and medical presentations in patients are presented in Table 3. To have a definitive identification, for all 77 isolates, *rpoB* gene sequencing was performed, which showed more than 99% homology with *M. abscessus* (n = 8/77, 10.38%), *M. simiae* (n = 18/77, 23.37%), *M. fortuitum* (n = 20/77, 25.97%), *M. chelonae* (n = 3/77, 3.89%), *M. kansasii* (n = 14/77, 18.18%) and *M. intracellulare* (n = 14/77, 18.18%). In Real-Time PCR, 69 isolates, which showed more homology with standard species were included: *M. abscessus* (n = 8/69, 11.59%), *M. simiae* (n = 16/69, 23.18%), *M. fortuitum* (n = 19/69, 27.53%), *M. chelonae* (n = 1/69, 1.44%), *M. kansasii* (n = 12/69, 17.39%)12 and *M. intracellulare* (n = 13/69, 18.84%). *M. fortuitum* was the most commonly isolated NTM in the present study by all applied methods. The results from ZNS and molecular methods are compared with culture. The applied phenotypic and molecular methods methods for EPNTM samples, demonstrated overall specificities, sensitivities, negative predictive, and positive predictive values (with 95% confidence intervals), which are indicated in Tables 4 and 5.

**Table 1:** Results from phonetypic tests and molecular methods for suspected NTM samples

Method	Extrapulmonary NTM* (N = 124)
AFB**	
Negative	95 (76.61%)
Positive	29 (23.38%)
**Culture & Biochemical tests**	
Negative	77 (62%)
Positive	47 (37.90%)
**Real-time PCR**	
Negative	55 (44.35%)
Positive	69 (55.64%)
***rpoB*** **sequencing**	
Negative	47 (37.9%)
Positive	77 (62%)

NTM*: non-tuberculous mycobacteria; AFB**: acid-fast bacilli

**Table 2:** The distribution of non-tuberculosis mycobacteria samples according to the patients’ sex and type of samples (n = 77)

EP NTM*	Female	Male	Total
lymph node	12	27	39
urine	1	2	3
skin lesion aspiration	5	5	10
pleural fluid	12	11	23
bone biopsy	1	1	2

EP NTM*: extrapulmonary non-tuberculous mycobacteria

**Table 3:** Baseline Characteristics of patients with EPNTM infection (n = 77)

Main symptoms	PMH*	EP NTM**
Productive cough, chest wall pain & weight loss	HIV^a^	26
Productive cough	Smoker	9
Local pain, small pale nodule	Respiratory failure	5
Productive cough, fever, body weight loss	Tuberculosis	11
Fever	HCV^b^	4
Fever, inflammation and tenderness in join	Diabetic	5
Productive cough, fever, weight loss	Immunocompromised	4
Local abscess and discharge	COPD^c^	13

PMH*: past medical history; EP NTM**: Extrapulmonay non-tuberculosis mycobacteria; HIV^a^: human immunodeficiency virus; HCV^b^: Hepatitis C virus; COPD^c^: chronic obstructive pulmonary disease

**Table 4:** Sensitivities and specificities of the two amplification systems of *rpoB* –based PCR sequencing and Real-time PCR (N = 124)

Type of sample and Method	Samples no.	Prevalence	95% CI^a^	Positive LR^b^	Negative LR
**lymph node (N = 54)**					
*rpoB* sequencing	39	72.22%	79–95	72.95	0.90
real-time PCR	33	61.11%	46–59	60.01	0.82
**urine(N = 20)**					
*rpoB* sequencing	3	0.15%	73–95	73.94	0.91
real-time PCR	1	0.05%	28–45	14.24	0.79
**skin lesion (N = 10)**					
*rpoB* sequencing	10	100%	70–95	100	0.00
real-time PCR	10	100%	70–95	100	0.00
**pleural fluid (N = 34)**					
*rpoB* sequencing	23	67.64%	69–95	79.97	0.95
real-time PCR	23	67.64%	33–54	79.27	0.95
**bone biopsy(N = 6)**					
*rpoB* sequencing	2	68.79%	62–95	79.95	0.99
real-time PCR	-	-	-	-	-

CL^a^: confidence interval, LR^b^: likelihood ratios

**Table 5:** Overall sensitivity, specificity, PPV and NPV of applied methods among positive samples for NTM (n = 77)

Method	Sensitivity % (95% CI)^a^	Specifiticy% (95% CI)	PPV^b^ %	NPV^c^ %
AFB*	29 (33–43)	34 (23–47)	34	53
Culture	100 (87–100)	100 (87–100)	100	99
NTM Real-time PCR	80 (72–89)	77 (62–88)	88	91
rpoB sequencing	100 (87–100)	100 (87–100)	100	98

AFB*: acid-fast bacilli, CI^a^: confidence interval; PPV^b^: positive predictive value; NPV^c^: negative predictive value

## Discussion

As the number of immunocompromised patients increases worldwide (such as cancer patients, transplant recipients, and those on immunosuppressive drugs), we are facing an increase in NTM infections (Pennington et al. 2021). The clinical manifestations of NTM disease are similar to those of TB and may pose a diagnostic challenge even to an experienced clinician (Sharma and Upadyay 2020). In endemic coutries for TB like Iran, NTM infections are frequently misdiagnosed as TB both from clinical manifestation and conventional laboratory criteria (Nasiri et al. 2018a, b). In this study, we investigated EP samples to characterize EPNTM diseases in patients from referral hospitals in southwest Iran. The rate of EPNTM were highest in Razi and Golestan main teaching hospitals as 55.84% (n = 43), and 44.15% (n = 34) respectively. We detected 62% (n = 77) NTM species among 124 EPNTM suspected specimens, these were mostly isolated from lymph node and pleural fluid specimens respectively, which accounts for the most prevalent obtained specimens in the present study. The results were not in concordance with other studies in developed and developing countries which they reported the prevalence of EPNTM lower in comparison to our study. Moreover in their studies, NTM lymphadenitis at 35.3% and peritoneal NTM at 12.05%, were reported as the frequent form of EPNTM (Fang et al. 2022; Abdallah et al. 2015). However, in the study performed by Sunnetcioglu et al. (2015), the main sites and rate of EPNTM involvement were lymph nodes (50%), and pleura (32%), which were in agreement with epidemiological data from our study. Moreover, similar to our findings, EPNTM was more commonly detected in mens in their study. The proportion of EPNTM infections has been growing over the past decades, with remarkable differences in the involvement organs and rates reported from different countries (Sama et al. 2016; Park et al. 2019). In this study the rate of EPNTM was (62%) which this rate was higher than to the prevalence of EPNTM in developing countries such as Turkey and Ethiopia at 49.4%, and 49.8% respectively (Sunnetcioglu et al. 2015; Arega et al. 2020). According to the Iranian ministry of health, EPNTM estimated 19.14% in Iran (Zahedi et al. 2017), and there has been decreasing in the rate since 2015 (Meghdadi et al. 2015; Hadifar et al. 2019). It was found that EPNTM patients were relatively young (mean age 32 years) and the proportion declined with age. Similar to our results, a study in Saudi Arabia reported a high prevalence of EPNTM in productive age groups (AlJumah et al. 2020). Because of the lack of adequate diagnostic facilities, complicacy of diagnosis, and absence of a national program, the importance of all forms of EPNTM is not recognized yet. Usually, histopathology examination of affected sites and tissues is recommended for diagnosing EPNTM patients. While the typical histopathological finding for EPNTM is a caseation granuloma, non-caseation granuloma may also begin TB treatment in our setting. To make such a decision, the high prevalence of TB and the lack of or non-availability of definitive diagnostics tools (e.g. the mycobacterial culture technique) need to be taken into account. We used a set of specific primers in PCR method for the detection of EPNTM, and 77 out of the total 124 suspected samples were positive. The results indicated that the PCR method revealed similar clinical sensitivity to culture as the standard gold method. In agreement with our results, the other studies showed that molecular methods are more sensitive than traditional methods. PCR technique can amplify different targets at the same time and it is used to detect and identify mycobacteria from the *M. tuberculosis* complex and NTM (Meghdadi et al. 2015). In current study we compared the sensitivity of both PCR and Real-Time PCR methods, and the results showed that PCR method reached a sensitivity of 100% but the sensitivity of Real-Time PCR was 80%. Recent studies, have so far assessed the Real-Time PCR sensitivity (Kalaiarasan et al. 2020; Wang et al. 2015), and in general, their sensitivity estimatation is higher than ours, perhaps because they dealt with a much higher proportion of smear-positive samples.

In conclusion, the magnitude of EPNTM can be overestimated for different reasons such as the fact that the study was on referred patients for TB to refrral hospitals. In addition, the diagnosis of most of the cases was not confirmed microbiologically and other mimic cases can be considered EPNTM. In general, the growing trend of EPNTM in Iran needs specific programs and resources to have a better diagnosis in Iran. PCR sequencing is a reliable method, it can be used to definitively confirm isolates with culture.

## Data Availability

All data generated or analyzed during this study are included in the present published article.

## Abbreviations

TB: Tuberculosis
PTB: Pulmonary tuberculosis
MTB: *Mycobacterium tuberculosis*
EPTB: Extrapulmonary tuberculosis
NTM: Nontuberculous mycobacteria
EPNTM: Extrapulmonary nontuberculous mycobacteria
HIV: Human immunodeficiency virus
ZNS: Ziehl–Neelsen staining
LJ: Lowenstein-Jensen
CDC: Centers for Disease Control and Prevention
MAC: *M. avium* complex
AFB: Acid-fast bacilli
dNTP: Deoxynucleotide triphosphate
NCBI: National Center for Biotechnology Information
WHO: World health organization.
